# Amyloid Presence in Acute Ischemic Stroke Thrombi: Observational Evidence for Fibrinolytic Resistance

**DOI:** 10.1161/STROKEAHA.124.050033

**Published:** 2025-05-27

**Authors:** Justine M. Grixti, Arun Chandran, Jan-Hendrik Pretorius, Melanie Walker, Alakendu Sekhar, Etheresia Pretorius, Douglas B. Kell

**Affiliations:** Department of Biochemistry, Cell and Systems Biology, Institute of Systems, Molecular and Integrative Biology, Faculty of Health and Life Sciences, University of Liverpool, United Kingdom (J.M.G., E.P., D.B.K.).; Department of Neurology, The Walton Centre, Liverpool, United Kingdom (A.C., A.S.).; Department of Economics (J.-H.P.), Stellenbosch University, South Africa.; Department of Physiological Sciences, Faculty of Science (E.P., D.B.K.), Stellenbosch University, South Africa.; Department of Neurological Surgery, University of Washington, Seattle (M.W.).; The Novo Nordisk Foundation Centre for Biosustainability, Technical University of Denmark, Kongens Lyngby (D.B.K.).

**Keywords:** amyloid, fibrin, humans, stroke, thrombectomy

In nearly one-third of patients with acute ischemic stroke (AIS), thrombolytic therapy fails to achieve vascular recanalization, and the reasons for this remain unclear.^1^ Recent research has highlighted the unique behavior of fibrin in forming an anomalous amyloid-like structure, termed fibrinaloid, that exhibits notable resistance to fibrinolysis.^2^ This fibrinaloid form has been observed predominantly in microthrombi (2–200 µm), raising questions about relevance in larger thrombi, such as those in AIS. This observational pilot study investigates whether thrombi retrieved during mechanical thrombectomy exhibit analogous amyloid characteristics, which could offer insights into thrombus persistence and resistance to lytics or natural degradation. Given the overlapping etiologies of AIS, we aimed to identify thrombus features that transcend subtype distinctions and may underlie shared resistance mechanisms.

Various stains and methods have been used to assess thrombus morphology, often to infer the origin and structure.^3^ However, to our knowledge, thioflavin T staining for amyloid in AIS thrombi has not been reported. Here, we used thioflavin T, a standard, reproducible amyloid stain, to detect amyloid content in thrombi extracted during mechanical thrombectomy.

Participants were selected from patients who underwent standard-of-care mechanical thrombectomy for AIS. Clots were obtained consecutively from patients who provided informed consent as part of the Walton Centre Clot Bank feasibility study (Integrated Research Application System project identifier 308223), the first stroke-specific thrombus biobank of the United Kingdom. Eight of 31 eligible patients consented to this analysis. Thrombectomies were performed under general anesthesia using flow arrest aspiration and/or stent retriever techniques, and thrombi were subsequently wax embedded using standard protocols. Retrieved thrombi were wax embedded, sectioned, de-waxed, and stained with 30 μmol/L thioflavin T, which fluoresces green upon binding amyloid fibrils, enabling robust and reproducible detection.

Of the 8 AIS cases analyzed, 2 were wake-up strokes with a last known normal to reperfusion time of ≈10 hours; the remaining 6 had a mean onset-to-recanalization time of 3 hours. The cohort had a mean age of 72.5 (SD, 10.3; range, 58–88) years, all identified as White, with 87.5% (7/8) men. Baseline functional status (modified Rankin Scale) ranged from 0 to 2 (median, 1), indicating generally good premorbid function. Six (75%) patients received alteplase before thrombectomy. Thrombus locations varied, most commonly involving the M1 segment of the middle cerebral artery. One thrombus (patient NB 23-226) involved a tandem cervical occlusion extending into the proximal internal carotid artery. All patients achieved successful reperfusion, with Thrombolysis in Cerebral Infarction 2B in 25% (2/8) and Thrombolysis in Cerebral Infarction 3 in 75% (6/8). Despite variation in thrombus location and technique, amyloid presence and extent were consistently high across all samples (Figure).

**Figure.**
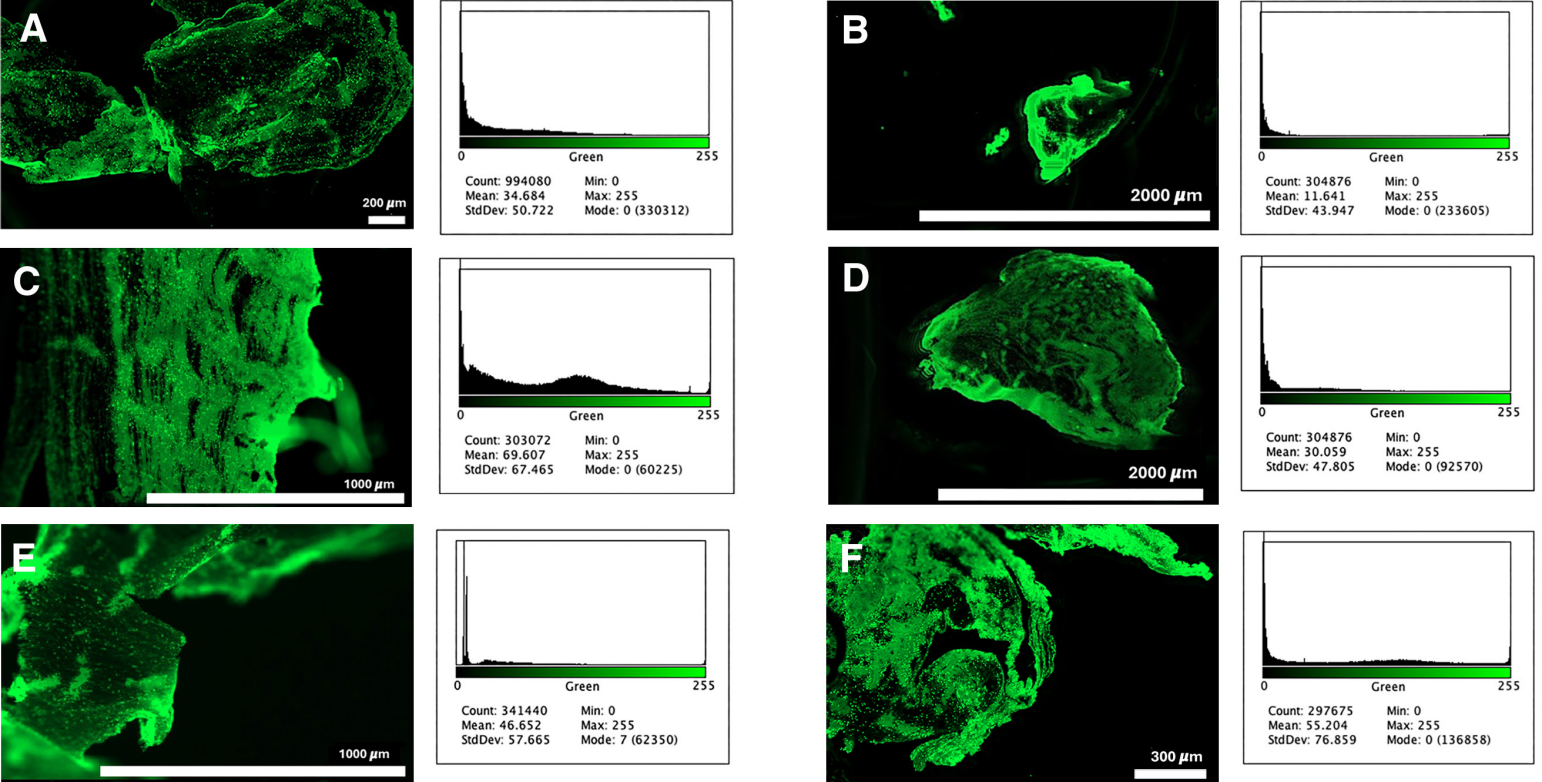
**Fluorescence images of thrombi from 8 patients with acute ischemic stroke (AIS), each stained with thioflavin T to indicate amyloid content.** Each part (**A**–**F**) represents a sample from a separate patient, with green fluorescence highlighting the presence and distribution of amyloid within the thrombi. The intensity and pattern of amyloid staining vary across the samples, reflecting heterogeneity in amyloid content and distribution. These images visually demonstrate the prevalence of amyloid within AIS thrombi, and its heterogeneity, which may contribute to their resistance to fibrinolysis.

Over a decade ago, electron microscopy revealed that AIS thrombi often exhibit a dense, matted structure, later identified as amyloid.^2^ Wax embedding has since been validated as a reliable medium for detecting amyloid via birefringent and fluorescent stains, minimizing artifact concerns. Our findings align with prior studies showing that Aβ (amyloid-β) binds fibrinogen, forming structurally altered clots. Amyloid-associated lytic resistance was observed in other vascular beds.^4^ These clots are characterized by thinner fibers and show increased resistance to fibrinolysis.^5,6^ Additionally, Aβ impairs the binding of plasminogen to fibrin, which reduces plasmin generation and consequently inhibits fibrinolysis.^5^ These mechanisms may explain the persistence of amyloid-laden thrombi in our cohort.

The high amyloid content observed may help explain thrombus genesis and persistence, particularly in fibrinolysis-resistant cases contributing to ischemic injury. Despite the small sample, consistent amyloid presence in all 8 cases supports the need for larger studies. These findings mark an important step toward establishing amyloid’s role in thrombus resistance and warrant further mechanistic investigation.

Interest in the amyloid composition of AIS thrombi is further supported by evidence that such thrombi may contain bacterial cell wall components.^7^ Even minimal concentrations of these products can catalyze fibrinaloid microthrombus formation,^2^ suggesting that amyloid accumulation may result from chronic inflammation or infection. The presence of amyloid in large thrombi raises questions about systemic or local inflammatory conditions promoting amyloidogenic fibrin changes. Systemic factors such as serum amyloid A may promote thrombus amyloidogenesis and impact lytic response.^8^ This suggests a possible mechanism whereby thrombi in patients with AIS could accumulate amyloid material over time, potentially due to chronic inflammation or infection.

Although this small cohort was not stratified by AIS subtype, the extensive (albeit heterogeneous) amyloid content within and between thrombi reveals a previously unreported AIS feature. This amyloid-rich composition may underlie resistance to fibrinolytics, whether localized to the thrombus shell or core.^9^ While preliminary, these insights suggest amyloid-targeted therapies could enhance AIS thrombolysis and merit further study. Findings from this study are available as a preprint.^10^

## Data Availability

All relevant data generated or analyzed during this study are included in this article. Findings from this study are available as a preprint.

## Statements and Declarations

This study was conducted with approval from the UK Health Research Authority and Research Ethics Committee (REC reference 22/NW/0087; Integrated Research Application System project identifier 308223). Thrombi were obtained via the Liverpool Stroke Clot Bank, based at the Liverpool Neuroscience Biobank, Walton Centre National Health Service (NHS) Foundation Trust. Amendments permitting sample transfer and fresh frozen collection were approved under REC 22/NW/0087/AM03 and AM04.

## Article Information

### Acknowledgments

This study is reported in accordance with the STROBE guidelines (Strengthening the Reporting of Observational Studies in Epidemiology) for cohort studies. The authors thank Drs Nitika Rathi and Piyali Pal for conducting preliminary analyses of the thrombi; Khaja Syed for establishing the clot bank and facilitating material transfer to Liverpool University; Sally Flintham for her support in thrombus collection and transfer; Helen Malone for assistance with patient consent; and Marie O’Brien for her expertise in microtoming and dewaxing at Liverpool Shared Research Facility (LIV-SRF) Histology, University of Liverpool.

### Sources of Funding

Funding was provided by the Balvi Foundation (grant 18), the Novo Nordisk Foundation (NNF20CC0035580), the National Research Foundation of South Africa (grant 142142), and the South African Medical Research Council (Self-Initiated Research [SIR] grant).

### Disclosures

Dr Pretorius is a named inventor on a patent application related to fluorescence methods for microclot detection in long COVID. D.B. Kell is on the Scientific Advisory Board of Daye, is a non-Executive Director of Innotivedx, and holds shares in these companies. The funders had no role in study design, data collection, analysis, interpretation, or publication decisions. The other authors report no conflicts.
